# Magnetic Nanoparticle-Based Dianthin Targeting for Controlled Drug Release Using the Endosomal Escape Enhancer SO1861

**DOI:** 10.3390/nano11041057

**Published:** 2021-04-20

**Authors:** Ajmal Zarinwall, Mazdak Asadian-Birjand, Didem Ag Seleci, Viktor Maurer, Alexandra Trautner, Georg Garnweitner, Hendrik Fuchs

**Affiliations:** 1 Institute for Particle Technology (iPAT), Technische Universität Braunschweig, 38104 Braunschweig, Germany; a.zarinwall@tu-bs.de (A.Z.); d.ag-seleci@tu-bs.de (D.A.S.); v.maurer@tu-bs.de (V.M.); 2 Center of Pharmaceutical Engineering (PVZ), Technische Universität Braunschweig, 38106 Braunschweig, Germany; 3 Charité–Universitätsmedizin Berlin, Corporate Member of Freie Universität Berlin and Humboldt-Universität zu Berlin, Institute of Laboratory Medicine, Clinical Chemistry and Pathobiochemistry, 13353 Berlin, Germany; mazdak.asadian-birjand@charite.de (M.A.-B.); alexandra.trautner@charite.de (A.T.); hendrik.fuchs@charite.de (H.F.)

**Keywords:** surface modification, functionalization, drug delivery, saponin, SO1861, EGF, targeted toxin, endosomal escape

## Abstract

Targeted tumor therapy can provide the basis for the inhibition of tumor growth. However, a number of toxin-based therapeutics lack efficacy because of insufficient endosomal escape after being internalized by endocytosis. To address this problem, the potential of glycosylated triterpenoids, such as SO1861, as endosomal escape enhancers (EEE) for superparamagnetic iron oxide nanoparticle (SPION)-based toxin therapy was investigated. Herein, two different SPION-based particle systems were synthesized, each selectively functionalized with either the targeted toxin, dianthin-epidermal growth factor (DiaEGF), or the EEE, SO1861. After applying both particle systems in vitro, an almost 2000-fold enhancement in tumor cell cytotoxicity compared to the monotherapy with SPION-DiaEGF and a 6.7-fold gain in specificity was observed. Thus, the required dose of the formulation was appreciably reduced, and the therapeutic window widened.

## 1. Introduction

Colorectal cancer is one of the leading causes of cancer-related deaths worldwide [1]. To date surgery is the conventional treatment, which is often accompanied by radiation- or chemotherapy. However, these supportive therapy methods lack specificity in the treatment of tumorous tissues, leading to severe toxic side effects and limited possible treatment doses. To overcome these drawbacks, the concept of targeted tumor therapy, which focuses on the development of novel adjuvant cancer treatments to solely target cancerous cells, stands out as a promising technology. For this purpose, specific targeted delivery vehicles, such as nanoparticle- or protein-based systems, have been designed [2,3]. The latter encompasses so-called targeted toxins—a group of therapeutics which comprise a toxic protein with enzymatic activity and a targeting moiety, addressing specific markers on the cellular surface of cancer cells. In particular, the epidermal growth factor receptor (EGFR) is known to be overexpressed in a variety of human cancer cell lines including pancreatic, breast, and colorectal cancers, and thus is an ideal marker for targeted tumor therapy [4,5]. Moreover, selectively addressing EGFR yields an optimized cellular internalization, which is crucial for improving the treatment efficacy and reducing the needed treatment dosage [6,7,8]. Nevertheless, the majority of macromolecular anti-tumor substances are internalized via receptor-mediated endocytosis [9,10], resulting in a pH-dependent degradation of the delivered therapeutics within the up-taking endosomes, and thus in a reduced effectiveness [11]. Consequently, a major bottleneck is the limited entry of the drug into the cytosol, as most of the delivered antitumor therapeutics are cytosolically active (macro-)molecules [12,13]. Thus, commonly high serum levels of the therapeutics are employed to overcome insufficient cytosolic entry and to ensure sufficient efficacy, resulting in severe side effects such as immunogenicity and vascular leak syndrome [14,15]. In order to circumvent this drawback, several systems have been studied to date as efficacy enhancers including the attenuation of the membrane integrity of endosomal membranes, disruption of endosomes, or utilization of cell penetrating peptides [16,17,18,19]. Notably, certain plant secondary metabolites (glycosylated triterpenoids) were shown to tremendously facilitate the endosomal escape, due to a destabilizing effect on the endosomal membrane while the plasma membrane remains unaffected, finally resulting in substantially improved anti-tumor activity of specific protein-based targeted toxins [20,21,22]. However, the specific mechanism of interaction between glycosylated triterpenoids and cellular membranes remains unclear, although several studies have been conducted to date [13,23]. In particular, the glycosylated triterpenoid SO1861, extracted from *Saponaria officinalis* L., was reported to exhibit this property when concomitantly administered with dianthin (Dia), a plant toxin from *Dianthus caryophyllus* L. [12,24,25]. Dia belongs to the class of ribosome-inactivating proteins (RIP) that cut off a specific adenine residue from ribosomal RNA, which finally results in the arrest of protein synthesis and apoptosis. However, for in vivo applications it needs to be taken into account that glycosylated triterpenoids are rapidly and unspecifically distributed inside an organism with disparate kinetics than Dia [26]. Moreover, the components, SO1861 and Dia, need to be delivered via two different routes, which limits the compatibility of the approach as a versatile platform [27]. Thus, for the realization of an augmented efficacy via enhanced endosomal escape mechanisms, the development of formulations for multi-component systems is of essential importance in order to synchronize the individual pharmacokinetics.

A promising approach to face these challenges is the use of nanomedicine, which focuses on the design of novel formulations for site-specific therapies. Superparamagnetic iron oxide nanoparticles (SPIONs) are highly promising for many nanomedical applications, as they have been shown to serve as a nanoscale carrier platform for various therapeutic agents because of their unique magnetic properties and their biocompatibility [28,29,30,31,32]. Furthermore, a suitable surface functionalization enables the defined coordination of different types of drugs and a tuning of the biochemical properties. Nonetheless, there are several problems of many common functionalization strategies, in particular the heterogeneity of the final product, the possibility of cross-linkage between particles, and the formation of oligomers at the particle surface. Furthermore, to render SPIONs viable for clinical translation, it is necessary to assure high efficacies to reduce the probability of side effects. To the best of our knowledge, no studies have yet been performed that examine the effect of glycosylated triterpenoid based endosomal escape enhancers (EEE) on the toxicity of RIP-modified nanoparticles on cancer cells.

**Figure 1:**
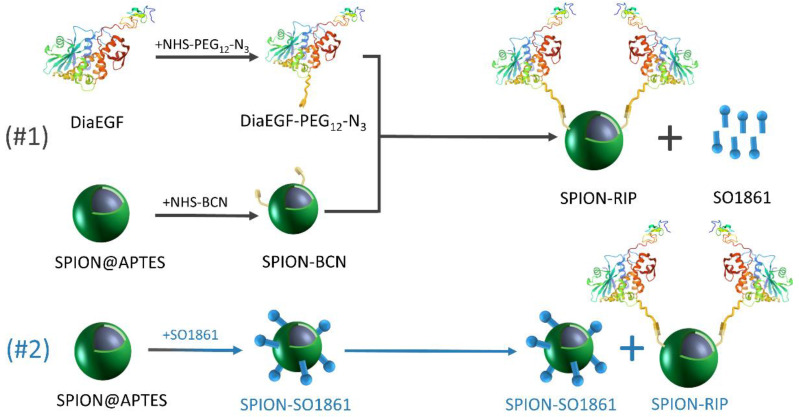
Schematic depiction of the synthesized particle systems including the individual experimental steps and the composition of the applied combinatorial approaches.

Herein, the suitability of SO1861 to act as an EEE for SPION-based Dia delivery systems was investigated. Therefore, we present a defined route using strain-promoted azide–alkyne cycloaddition “click-chemistry” for selectively functionalizing SPIONs with Dia as well as EGFR-targeted dianthin (Dianthin-EGF; DiaEGF). The obtained particle systems were characterized by several methods, such as transmission electron microscopy (TEM), thermogravimetric analysis (TGA), and dynamic light scattering (DLS). The efficacy of Dia and DiaEGF after immobilization on the SPION surface was tested in vitro on colorectal cancer cells. Furthermore, the ability of SO1861 to enhance the efficacy of RIP-functionalized SPIONs was investigated in two combinatorial approaches (Figure 1 and Figure S1). At first, SO1861 was added as a bare solution to the particle systems. Secondly, SO1861 was conjugated separately on SPIONs and added as a particle mixture together with RIP-functionalized SPIONs to the cells in order to unify the pharmacokinetics of the RIPs and SO1861. 

## 2. Materials and Methods

### 2.1. Chemicals and Reagents

Acetone (≥99.9%), (3-aminopropyl)triethoxysilane (APTES, 99%), (1R, 8S, 9s)-bicyclo[6.1.0]non-4-yn-9-yl-methyl-*N*-succinimidyl carbonate (BCN-NHS), dimethylformamide (DMF, ≥99.8%, A. C. S. reagent), iron(II) chloride tetrahydrate (FeCl_2_∙4H_2_O, ≥99%), iron(III) chloride hexahydrate (FeCl_3_∙6H_2_O, ≥99%), 4-(2-hydroxyethyl)-1-piperazineethanesulfonic acid (HEPES, ≥99.5%), hydrochloric acid (37%), sodium hydroxide (NaOH, ≥97%), 3-(4,5-dimethylthiazol-2-yl)-2,5-diphenyl tetrazoliumbromide (MTT, 98%), ammonium hydroxide solution (NH_4_OH, 28.0–30.0%), and triethylamine (TEA, ≥99.5%) were obtained from Sigma-Aldrich Chemie GmbH (Taufkirchen, Germany). Azido-PEG_12_-NHS ester (N_3_-PEG_12_-NHS, >95%) was acquired from Conju-Probe LLC (San Diego, CA, USA). 1-[Bis(dimethylamino)methylene]-1H-1,2,3-triazolo[4,5-b]pyridinium 3-oxide hexafluorophosphate (HATU) was purchased from Santa Cruz Biotechnology, Inc. (Dallas, Texas, USA) Hydrogen peroxide solution (30%, A. C. S. reagent) was supplied by Merck KGaA (Darmstadt, Germany). Ethanol (HPLC grade) and dimethyl sulfoxide (DMSO, HPLC grade) were bought from Fisher Scientific GmbH (Munich, Germany).

Unless otherwise specified, de-ionized water was used. However, the HEPES buffer was prepared as a 10 mM solution using Milli-Q water. The pH was adjusted to 7.4 by 1 M NaOH solution.

### 2.2. SPION Synthesis and APTES Modification

The SPIONs were synthesized and modified with APTES (SPION@APTES) in a one-step co-precipitation method. In a typical synthetic procedure, 0.75 g of FeCl_3_∙6H_2_O and 0.375 g of FeCl_2_∙4H_2_O were dissolved in 50 mL de-ionized water (dH_2_O) in a 100 mL three-necked round bottom flask. The solution was refluxed at 80 °C and stirred for 10 min under nitrogen atmosphere. Then, 10 mL of NH_4_OH (28–30% aqueous solution) was injected dropwise, via a syringe to the solution. After one hour, 15 mL of EtOH, followed by a previously prepared solution of 2 mL of APTES in 25 mL EtOH, was added to the reaction mixture and stirred for further 3 h at 80 °C. Prior to the purification, the suspension was allowed to cool to room temperature (RT). Then, as-synthesized nanoparticles were retrieved from the mixture by the addition of acetone (5:1 *v*/*v* acetone/supernatant) as precipitant, followed by centrifugation for 10 min at 8500 rpm, resuspended in 20 mL dH_2_O, and dialyzed against 2 L dH_2_O for 24 h. The resulting magnetic nanoparticle concentration of 26.67 mg/mL was determined gravimetrically.

### 2.3. BCN Modification

To implement selective binding sites between the RIPs and the particle surface, SPION@APTES was first modified with BCN-NHS (SPION-BCN) according to Asadian-Birjand et al. [33], with slight modifications. 376 μL of the aqueous SPION@APTES suspension (10 mg SPIONs) and 50 μL of TEA were diluted with 20 mL DMF. Thereafter, a twofold molar excess of BCN-NHS (1.02 mg), with respect to the peripheral amine moieties of SPION@APTES, was added and the reaction was carried out for 18 h at RT on a shaking device. The molar quantity of the grafted amine moieties per g_SPIONs_ was calculated from TGA measurements according to Equation (2) (see Section 2.11). Afterwards, the particles were centrifuged for 10 min at 8500 rpm and the supernatant was replaced with HEPES buffer for three times.

### 2.4. Expression and Purification of Dia and DiaEGF

Purification and expression of ^His^dianthin-30, hereafter simply referred to as Dia, and DiaEGF was performed as reported previously [34,35]. Briefly, plasmids (His^6^-tagged-dianthin-30-pET11d and His^6^-tagged-dianthin-EGF-pET11d) were transformed into *Escherichia coli* NiCo21(DE3) competent cells (Novagen, San Diego, CA, USA). To induce the expression of the RIPs, isopropyl β-D-1-thiogalactopyranoside (IPTG) was added at a final concentration of 1 mM. After 3 h of incubation at 37 °C and 120 rpm, bacterial suspensions were lysed by sonication with an ultrasound device (Branson Sonifier 250, G. Heinemann, Schwäbisch Gmünd, Germany). Purification of the released His^6^-tagged-toxins was achieved by nickel nitrilotriacetic acid chromatography (Ni-NTA Agarose; Qiagen, Hilden, Germany) followed by chitin column affinity chromatography, which served to remove bacterial proteins with binding activity for Ni-NTA agarose. Afterwards, the purity of the recombinant proteins was analyzed by sodium dodecyl sulfate-poly acrylamide gel electrophoresis (SDS-PAGE, 12% [*w*/*v*] gel) and the concentration was determined by a bicinchoninic acid (BCA) assay (Pierce, Rockford, IL, USA).

### 2.5. Modification of Dia and DiaEGF

To bind the RIPs specifically to the particles, and thus prevent crosslinking reactions, the toxins were first provided with the azide click ligand NHS-PEG_12_-N_3_ for a copper free strain-promoted alkyne-azide cycloaddition. Therefore, and to study the conjugation efficiency of the NHS-PEG_12_-N_3_ linker to the toxins, three different molar ratios (1.5, 3.0, 20 mol_linker_/mol_toxin_) were employed. 0.3 mg of the respective RIP was mixed with the correspondent amount of the PEG linker and incubated for 3 h at 20 °C and 800 rpm in Dulbecco’s phosphate-buffered saline buffer. The conjugates were washed three times with the same buffer by means of Amicon centrifugal filter devices (10 kDa; Merck Millipore, Burlington, MA, USA). Successful conjugation was confirmed by matrix-assisted laser desorption ionization time of flight mass spectrometry (MALDI-TOF-MS), which was conducted on an Ultraflex-III TOF/TOF instrument (Bruker Daltonics, Billerica, MA, USA) equipped with a 200 Hz N_2_-laser with 337 nm wavelength (pulse energy of 150 µJ) and 3 ns pulse width, and operated in the positive linear mode.

### 2.6. Determination of Enzymatic Activity

*N*-glycosidase activity of RIPs was determined by an adenine release assay which is based on the cleavage and release of adenine residues from herring sperm DNA [25]. First, an adenine standard curve was prepared by determining the absorbance at 260 nm for different adenine concentrations (10, 20, 40, 80, 160, and 320 pmol/μL). A NanoDrop ND-1000 spectrophotometer (PEQLAB Biotechnologie GmbH, Erlangen, Germany) was used to measure the absorbance. Afterwards, a herring sperm DNA stock solution (5 μg/μL; Invitrogen, Carlsbad, CA, USA) was prepared using a 50 mM sodium acetate buffer (pH 4, 100 mM KCl) and preheated at 50 °C for 10 min. Then, 20 μL of the stock solution was added to 30 pmol of the unmodified and modified RIPs and diluted to a total volume of 50 μL by addition of acetate buffer. The reaction was then carried out at 50 °C for 1 h under continuous shaking. Subsequently, the released adenine was separated by centrifugation with filter membranes (3000 MWCO; Merck Millipore, Burlington, MA, USA) for 45 min at 4 °C and 5000× *g*. Finally, the absorbance of the flow-through was measured at 260 nm. Quantification of adenine release was calculated by the use of the adenine calibration curve.

### 2.7. RIP Conjugation to SPIONs (SPION-Dia, SPION-DiaEGF)

0.75 mg of the respective modified RIP (Dia or DiaEGF) was added to 5 mg SPION-BCN in 15 mL HEPES buffer and incubated at RT for 20 h on a shaking device. The subsequent purification was carried out by repeated magnetic precipitation of the particles, exchanging the supernatant for three times. In addition, a negative control ensured that unconjugated RIPs were efficiently removed during subsequent washing and dispersing steps. For this purpose, 0.75 mg of DiaEGF was added to 5 mg SPION@APTES without prior modification by BCN-NHS and N_3_-PEG_12_-NHS, respectively. Incubation and purification were carried out under same conditions as mentioned in the section of BCN modification.

### 2.8. SO1861 Isolation

SO1861 was isolated from *Saponaria officinalis* L. caryophyllacea by methanol extraction of the powdered plant roots, acetone precipitation, and high performance liquid chromatography, as reported elsewhere [36]. Purity and identity of SO1861 were analyzed via LC/MS with an Agilent 6210 TOF LC/MS system.

### 2.9. Synthesis of SPION-SO1861

Coupling of SO1861 to the SPION surface (SPION-SO1861) was achieved using HATU as a zero-length crosslinker. Therefore, HATU and SO1861 were each first dissolved in DMSO with a concentration of 1 mg/mL. Then, 0.83 mL of HATU was added to 0.812 mL SO1861 and reacted for 20 min at RT and 800 rpm. Afterwards, activated SO1861 was added to 1 mg of SPION@APTES in 3 mL DMSO. The reaction was incubated for 18 h at RT and subsequently washed three times by centrifugation for 12 min at 8500 rpm with HEPES buffer. Analogous to the protein conjugation, non-activated SO1861 served as control to validate the purification efficiency. Thus, the reaction was repeated without prior activation of SO1861 by HATU. The experimental setup, as well as the washing procedure was carried out as described for the SPION-APTES modification.

### 2.10. Cell Culture

HCT-116 (colorectal carcinoma) and MDA-MB-453 (breast carcinoma) were cultured in Roti^®^-CELL McCoy’s 5A and Leibovitz’s L-15 medium, respectively, supplemented with 10% fetal bovine serum (BioChromKG, Berlin, Germany) and 1% penicillin/streptomycin (Gibco/Invitrogen, Karlsruhe, Germany). HCT-116 cells overexpress EGFR on their cellular surface and were grown in humidified incubators at 5% CO_2_ and 37 °C [37]. In contrast, MDA-MB-435 cells do not express EGFR and thus served as off-target cells, which were cultivated at 37 °C without CO_2_ [38].

Cytotoxicity studies were essentially carried out as described in past publications using an MTT assay [35]. Cells were seeded (3000 cells/well for HCT-116 and 30,000 cells/well for MDA-MB-453) on a 96-well plate in 100 μL medium and grown for 24 h. 80 μL of media and 20 μL HEPES buffer containing different iron concentrations (up to 2 mM Fe^3+^ final concentration) were added. Controls were provided with only 20 μL HEPES buffer. Regarding the combinatorial approach with free SO1861, cells were first incubated with 0.5 μg/mL SO1861 in 80 μL media for 15 min at RT and subsequently treated with SPIONs, suspended in 20 μL HEPES buffer. For the second combinatorial approach with conjugated SO1861, SPION-RIPs and SPION-SO1861 were mixed together and applied in 20 μL HEPES buffer at the same time. All cytotoxicity studies were carried out for 48 h. Afterwards, the media was aspirated and 200 μL of fresh media and 30 μL of a 5 mg/mL MTT solution were added and incubated for further 2 h at 37 °C. Thereafter, the supernatant was removed again, and formazan crystals were dissolved with 50 μL DMSO for 15 min on a shaker. Prior to the measurement, remaining particles were removed by placing a flat magnet under the plate and transferring the supernatant to another 96-well plate. The absorbance was measured at 570 nm (reference at 630 nm) by the SpectraMax 340PC Absorbance Microplate Reader (Molecular Devices, Sunnyvale, CA, USA). The relative cell viability related to control samples was calculated and as a result, the characteristic half-maximum inhibitory concentration (*IC_50_*) was determined as a quantitative measure. Furthermore, the enhancement factor (*EF*) for both combinatorial approaches was determined using the *IC_50_*-value of SPION-RIP (*IC_50_*_,-SO1861_) and the *IC_50_*-value after additional application of the respective SO1861 formulation (*IC_50_*_,+SO1861_):

(1) $$EF=\frac{IC_{50\left(+\text{SO1861}\right)}}{IC_{50(-\text{SO1861}})}\;$$

### 2.11. Characterization of SPIONs

The morphology and size of the obtained SPIONs were studied by transmission electron microscopy (TEM) using a Tecnai G^2^ F20 TMP from FEI at 200 kV. The particles were deposited on a carbon film of a 3.05 mm woven copper net with 300 mesh from Plano GmbH. Particle sizes were determined by measuring 50 nanoparticles, using the ImageJ software (Version 1.42q). Hydrodynamic diameter and size distribution measurements were performed on a Zetasizer Nano-ZS from Malvern Instruments (Kassel, Germany) by dynamic light scattering (DLS). All samples were characterized as aqueous suspensions for three times with a 173° backscatter geometry at 25 °C. Zeta potentials were evaluated by the same device using a capillary zeta cuvette (DTS1070C, Malvern Panalytical Ltd). Qualitative information on the molecules linked to the particle surface was obtained by FTIR spectrometry. Therefore, dried samples were analyzed with a Bruker Vertex 70, equipped with an attenuated total reflectance (ATR) cell. Thermogravimetric analysis (TGA) was carried out using a TGA/DSC 1 STAR^e^ system and a gas controller 4C200 STAR^e^ system from Mettler Toledo. 10 mg of the dried sample was heated at a rate of 10 °C/min under an oxygen atmosphere. Based on the recorded thermograms, the molar concentration of chemisorbed ligand (*c_ligand_*) per g_SPION_ was calculated [39]:

(2) $$c_{ligand}=\frac{\left((\Delta m_{\mathrm{mod}}-\Delta m_{unmod}\right)/100)}{M_{ligand}}\;\mathrm{given}\;\mathrm{in}\;{\mathrm{mol}}_{\mathrm{ligand}}*g_{\mathrm{SPION}}^{-1}$$

with Δ*m_mod_* and Δ*m_unmod_* being the mass loss of modified and unmodified SPIONs in percent, respectively, and *M_ligand_* being the molecular mass of the ligand. The number of molecules grafted on the SPION surface per square nanometer (referred as grafting density *δ_ligand_*) was then calculated as:

(3) $$\delta_{ligand}=\frac{c_{ligand}*N_{A}}{S_{A}*10^{18}}\;\mathrm{given}\;\mathrm{in}\;\mathrm{molecules}*{\mathrm{nm}}^{-2}$$

where *N_A_* is the Avogadro number and *S_A_* the specific surface area in m^2^ g_SPION_^−1^. *S_A_* was determined via nitrogen sorption measurements on the unmodified nanoparticles using the multipoint Brunauer, Emmett, and Teller (BET) surface analyzer ASAP 2460 from Micromeritics (Norcross, GA, USA). Therefore, the particles were first degassed under a vacuum for 2 h at 120 °C.

T_1_ and T_2_ relaxation times and relaxivities were determined on a TD-NMR analyzer at 0.94 T (minispec nq40 NMR analyzer, Bruker, Billerica, MA, USA). The values were measured at 40 °C in water at different iron concentrations.

## 3. Results and Discussion

### 3.1. Expression, Purification and Modification of Dia and DiaEGF

After recombinant expression in bacteria, Dia and DiaEGF were purified by Ni-NTA and chitin column affinity chromatography. Comprehensive analyses of the expression and purification of Dia and DiaEGF were carried in our previous studies [24,34,40] and will not be discussed in detail here. However, both RIPs, Dia and DiaEGF, appear as a single band in the SDS-PAGE (Figure S2) at approximately 30 kDa and 36 kDa, respectively, and thus no degradation products were observable. The obtained masses of Dia and DiaEGF were confirmed via MALDI-TOF-MS measurements in positive linear mode (Figures S3 and S4). For the processing of the final anti-tumor particle system, it is substantial to maintain a high enzymatic activity of the RIPs and a functional EGF binding domain in DiaEGF. This is accomplished by ensuring that RIPs are specifically bound to the SPION surface without uncontrolled crosslinking reactions. To this end, Dia and DiaEGF were first decorated with the heterobifunctional NHS-PEG_12_-N_3_ linker by amide bond formation between the NHS group of the linker and the lysine moieties of the proteins. To enable a precise adjustment of the conjugation density, three different feed ratios of linker to RIP were explored and analyzed by MALDI-TOF-MS (Figures S3 and S4).

**Figure 2:**
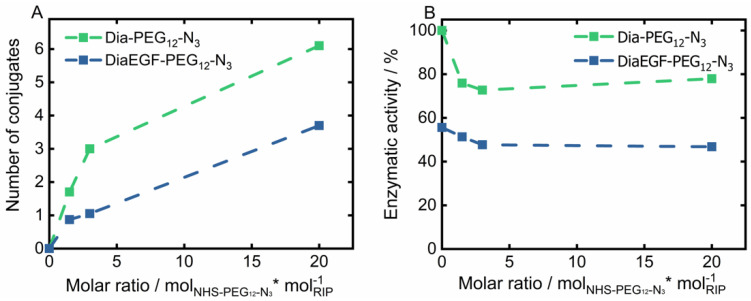
(**A**) Total amount of conjugated NHS-PEG_12_-N_3_ linker on Dianthin (Dia) and DianthinEGF (DiaEGF) and (**B**) the corresponding enzymatic activity in dependence of the initially used molar ratio of linker to the respective ribosome-inactivating protein (RIP; Dia or DiaEGF). Activity results are shown relative to the activity of unconjugated Dia, which was set to 100%.

The derived conjugation efficiency is shown in Figure 2A. Accordingly, the maximum number of conjugable linker molecules is restricted by the amount and accessibility of lysine groups present in the tertiary structure of the RIPs. A 20-times molar excess of linker to RIP resulted in the attachment of six and four linker molecules per Dia and DiaEGF, respectively. It is worthwhile to note that even though Dia contains 19 and DiaEGF 21 lysine moieties [25,40], only a small fraction of the linker molecules could be attached, showing that their accessibility is of utmost importance. A slightly larger number of linkers were conjugated to Dia than to DiaEGF, which can be ascribed to the occupancy of potentially accessible lysine residues by the fusion of EGF to Dia. To ensure that the anti-tumoral efficiency of the conjugates was not decisively declining, the *N*-glycosidase activity of the RIP domain was determined using an adenine release assay (Figure 2B). Accordingly, 203.4 pmol adenine/pmol toxin/h (100%) was released by ligand-free Dia, compared to 113.0 pmol adenine/pmol toxin/h in case of DiaEGF. Thus, 44.4% loss in catalytic activity occurred, which is commonly observed when RIPs are expressed as targeted toxins [25,34,40,41]. In contrast, conjugation of NHS-PEG_12_-N_3_ to Dia or DiaEGF only caused a moderate decrease in the enzymatic activity of approximately 25 and 9%, respectively. Interestingly, no significant differences were observed when the number of conjugated linkers was increased, indicating that lysines located close to the active center are always included in the conjugation reaction or do not affect the activity when conjugated with a linker that is rather small, compared to EGF. Nevertheless, all conjugates were still highly active and thus could be utilized for further processing. In order to facilitate the reaction of RIPs towards the SPION surface, RIP conjugates with the largest number of bound linkers were used for subsequent particle functionalization. Since SPIONs were functionalized with an excess of BCN compared to the subsequently added amount of RIP conjugates, the potential number of bound RIPs should not be limited even if one RIP reacts with multiple BCN linker molecules on the particle surface.

### 3.2. Characterization of Synthesized and RIP-Functionalized SPIONs

**Figure 3:**
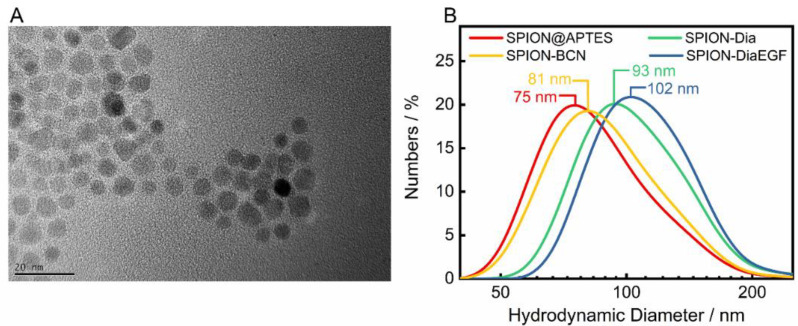
(**A**) Representative TEM image of SPION@APTES and (**B**) dynamic light scattering (DLS) results including the respective median sizes.

SPIONs were utilized as delivery platforms for Dia and DiaEGF, as well as for SO1861. For an understanding of the precise effect of the RIPs and SO1861 after their conjugation to the SPIONs, it is indispensable to thoroughly characterize the as synthesized and subsequently functionalized particle systems. The recorded X-ray diffraction (XRD) pattern of the as synthesized SPIONs is depicted in the Figure S5. Applying the Debye–Scherrer equation to the highest intensity reflection at 35.5° with a full width at half maximum of 0.7°, K_S_ of 0.9 and wavelength λ of 0.154 nm, a crystallite size of 12.8 nm was determined. This is in good agreement with the mean particle size of 11.9 ± 2.5 nm analyzed by TEM (Figure 3A), considering the measurement uncertainties. Furthermore, a relatively uniform spherical morphology of the SPIONs was obtained. As expected, the particle sizes measured by DLS (Figure 3B) were larger than the ones determined by TEM, since DLS takes into account not only the particle core, but also the APTES coating, as well as the hydration shell. A hydrodynamic diameter of 75 nm for SPION@APTES suggests that smaller SPION aggregates were silanized rather than individual particles. Subsequent conjugation of BCN and Dia or DiaEGF resulted in a further increase in hydrodynamic size by 6, 18, and 27 nm, respectively, indicating the additional presence of these larger ligands at the SPION surface.

**Figure 4:**
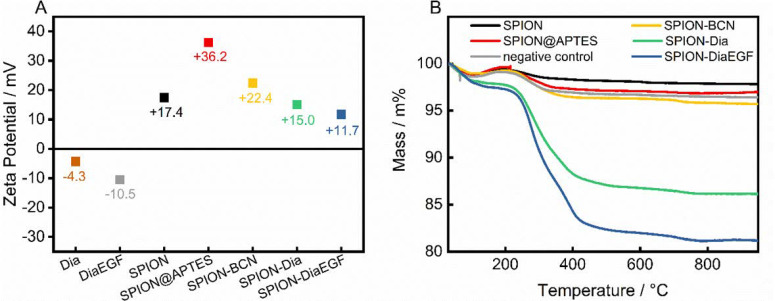
(**A**) Zeta potential measurements and (**B**) obtained thermograms after each functionalization step.

In addition, the zeta potential of the individual precursors and the particle systems after each functionalization step was measured (Figure 4A). After APTES modification, SPION@APTES shows a highly positive zeta potential. Successive functionalization leads to a severe shielding of surface charges in addition to higher particle volumes. Both result in a declining electrophoretic mobility and hence a decrease of the zeta potential. Nevertheless, the particles are not subjected to a significant tendency to agglomerate, as shown in the DLS curves—presumably due to steric stabilization.

To determine the amount of successfully conjugated ligand, thermal analyses were carried out (Figure 4B). The thermogram of pristine SPIONs shows a loss of 1.2 wt% within the ramp to 105 °C, as a result of water desorption. Upon further heating, a slight increase in mass by 0.6 wt% was noted, which is related to the oxidation of magnetite to maghemite [42], followed by a continuous decline of 1.6 wt%, due to decomposition of surface-bound organic compounds. In contrast, SPION@APTES exhibit a total mass loss of approximately 3.1 wt%. With S_A_ determined as 83 m^2^/g_SPION_, the grafting density of APTES can be calculated to 1.3 molecules_APTES_/nm^2^ using Equations (2) and (3). According to our previous study [39], this can be correlated with the formation of a homogenous monolayer, which is important for achieving the highest possible grafting density in addition to an evenly distributed surface coverage. Immobilization of Dia or DiaEGF leads to a greater mass loss of 13.9 and 18.8 wt%, respectively. The larger value for DiaEGF can mainly be attributed to its larger molecular mass (36,182 Da) compared to Dia (29,666 Da). Since FTIR spectroscopy did not reveal significant differences that can be used to verify the linkage between RIPs and SPIONs (Supplementary Information, Figure S6), a negative control was applied without utilizing the click ligands required for a covalent conjugation between RIP and SPION. The recorded total mass loss accounts for only 3.6 wt%, which ensures that unreacted RIPs are efficiently removed in the standard purification steps of the functionalization procedure, and thus, the greater mass losses obtained for SPION-Dia and SPION-DiaEGF can solely be attributed to chemisorbed RIPs.

### 3.3. Synthesis of SPION-SO1861

**Figure 5:**
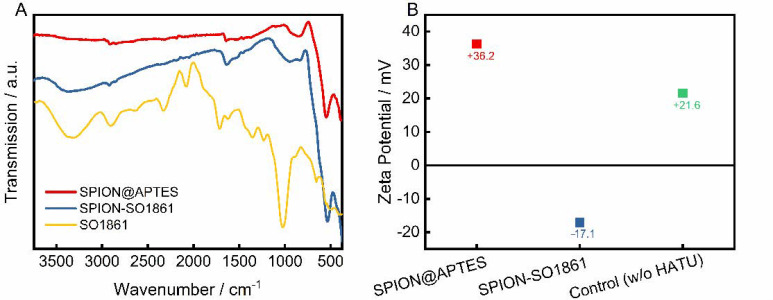
(**A**) FTIR-spectra and (**B**) zeta potential measurements before and after functionalization with SO1861. In the control experiment SPION@APTES and SO1861 were mixed together without the addition of HATU.

SO1861 was separately coordinated to SPION@APTES as a supplementary particle system. The successful binding was confirmed by FTIR and zeta potential measurements, as shown in Figure 5. The FTIR spectra (Figure 5A) of SPION@APTES and SPION-SO1861 show strong absorptions attributable to the Fe–O bonds at 535 cm^−1^. Furthermore, a small peak at 1650 cm^−1^ is apparent, which can be attributed to the bending mode of free amino (–NH_2_) groups. Because of the formation of amide bonds associated with the carbonyl (–C=O) stretching vibration at 1654 cm^−1^, the peak becomes significantly stronger for SPION-SO1861 [43]. In addition, the appearance of a new broad band at 3390 cm^−1^ in the spectrum of SPION-SO1861 can be ascribed to hydroxyl (–O–H) groups present in the sugar moieties of SO1861, which indicates the presence of SO1861 on the particle surface. The strong CO_2_ absorption bands at 2000 and 2150 cm^−1^ in the spectrum of free SO1861 result from system-related measurement interferences and are thus not relevant. As a result of SO1861 binding to the amino-terminated particle surface, the hydrodynamic diameter of SPION@APTES increased slightly from 75 nm (Figure 3B) to 84 nm (Figure S7). Furthermore, a change in the surface properties was also registered by analysis of the electrophoretic mobility (Figure 5B). Accordingly, the zeta potential significantly shifted from +36.2 mV to −17.1 mV, due to the binding of SO1861. In contrast, the zeta potential remained highly positive in the control experiment in which SPION@APTES was mixed with bare SO1861. In the control experiment, no chemical reaction was expected to occur between the SPION@APTES and SO1861, as the carboxylic acid group of SO1861 was not activated by HATU. In summary, both the FTIR and zeta potential measurements strongly indicate a successful conjugation of SO1861 to SPION@APTES.

### 3.4. In Vitro Cytotoxicity Studies

**Figure 6:**
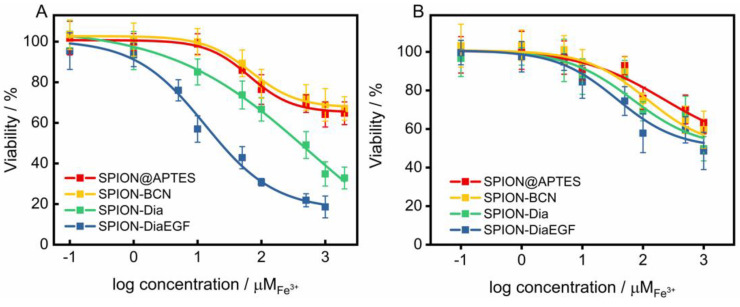
(**A**) Cytotoxicity studies of each particle system on epidermal growth factor receptor (EGFR)-overexpressing (EGFR/+) HCT-116 cells and (**B**) on MDA-MB-453 cell line which served as EGFR negative control (EGFR/−). Cell viability was assessed after 48 h by MTT-assay and plotted as a function of the molar iron concentration. Each point represents the mean ± SD of at least 3 independent experiments, performed in quadruplicate. Corresponding half maximal inhibitory concentration (*IC50*) and enhancement factor (EF) values are shown in Table 1.

As shown above, the enzymatic activity of Dia and DiaEGF was either affected by fusion of EGF to Dia or by attaching linker molecules to the RIPs. However, control experiments of free Dia-PEG_12_-N_3_ and DiaEGF-PEG_12_-N_3_ conjugate (Figure S8) on the target cells HCT-116 revealed that the RIP conjugates are still highly efficacious when administered to the cells. Thus, in order to finally evaluate the functionality of Dia and DiaEGF after their binding to SPION, the cytotoxicity and thus tumor cell therapeutic potential of the complete particle systems was investigated in detail. Furthermore, the influence of SPION@APTES and SPION-BCN on the cells was evaluated to assure that the toxic effects are solely related to the bound RIPs. As depicted in Figure 6A and Table 1, both SPION@APTES and SPION-BCN reduced the cell viability of the target cells HCT-116 to only 65% in the examined concentration range. Hence, it was not possible to calculate the specific concentration of particles at which the cell growth was inhibited by 50% (*IC50*). However, particles functionalized with Dia or DiaEGF exhibited a severe dose-dependent decrease in cell viability. Thus, *IC50* values of 5.5 × 10^−4^ and 1.3 × 10^−5^ M Fe^3+^ were recorded for SPION-Dia and SPION-DiaEGF, respectively. Since a reduced *IC50* value and thus a higher cytotoxicity can be related to a greater accumulation of particles within the cells, the obtained data verifies that the EGF binding domain of DiaEGF remained intact and was sterically not effectively hampered by the conjugation on the particle surface to act as targeting moiety. Since the employed surface chemistry for the linkage of the RIPs on the particle surface results in a covalent bond, non-specific leakage of Dia or DiaEGF can be neglected. Consequently, having in mind that Dia is only active in the cytosol, it can be assumed that more particles are internalized into the cells. The targeting effect of EGF can be quantified as the ratio of the *IC50* values of SPION-Dia to SPION-DiaEGF, which was 42, meaning that the targeting moiety provides a 42-fold increase in efficacy. The specificity of SPION-DiaEGF towards EGFR-overexpressing cells was successfully validated by concomitantly applying the particles on the non-target cell line, MDA-MB-453 (Figure 6B). In contrast to the target cell line, HCT-116, the toxic profiles of SPION-Dia and SPION-DiaEGF on the non-target cell line differ only slightly. The marginally higher cytotoxicity of SPION-DiaEGF may be ascribed to the non-specificity of the internalization process of non-target cells, resulting in broad variation of ingested particles in addition to a remaining level of low EGFR expression on the cellular membrane of MDA-MB-453 [44]. Thus, the obtained data in Figure 6A,B suggest that internalization of SPION-Dia in general is primarily achieved by unspecific mechanisms, such as pinocytosis, whereas SPION-DiaEGF seems to be ingested via receptor-mediated endocytosis, which is further supported by the observed enhancer effect of SO1861 (see below).

**Figure 7:**
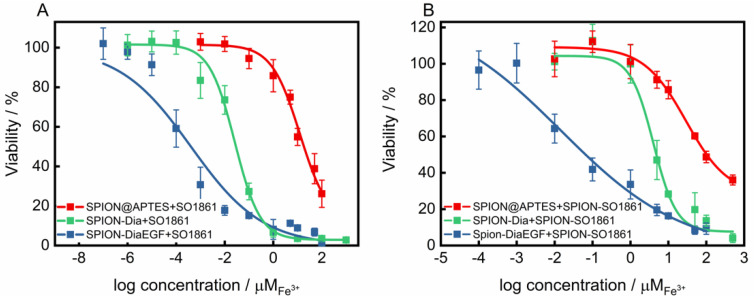
Cytotoxicity studies of SPIONs after each functionalization step on EGFR-overexpressing HCT-116 cells (**A**) applied in a combinatorial approach with 0.5 μg/mL free SO1861 and (**B**) with SPION-SO1861, respectively. Cell viability was assessed after 48 h by MTT-assay and plotted as a function of the molar iron concentration. Each point represents the mean ± SD of at least 3 independent experiments, performed in quadruplicate. Corresponding *IC50* and EF values are shown in Table 1.

The suitability of SO1861 to facilitate the endosomal escape and synergistically enhance the toxicity of RIP loaded SPION, was validated in two combinatorial approaches. First, the self-cytotoxicity of SO1861 on HCT-116 was evaluated (Supplementary Information, Figure S9). Accordingly, this compound presented no cytotoxicity up to 2 μg/mL. Nevertheless, a concentration of 0.5 μg/mL was considered as non-toxic for the combinatorial cytotoxicity experiments. RIP-loaded SPIONs in combination with free SO1861 (SPION-RIP+SO1861) were highly toxic in a synergistic fashion (Figure 7A). The resulting *IC50* values (Table 1) were interpolated to 2.6 × 10^−8^ M Fe^3+^ and 4.0 × 10^−10^ M Fe^3+^ considering SPION-Dia and SPION-DiaEGF, respectively, corresponding to a targeting effect of EGF of 65, which is slightly better than in the absence of SO1861. Consequently, a cytotoxicity enhancement factor (EF) of around 21,000- and 33,000-fold was achieved for SPION-Dia and SPION-DiaEGF. It is known that glycosylated triterpenoids also enhance the cytotoxicity of non-targeted RIPs as long as such RIPs reach the endosomes [45], which appears to be partially the case for SPION-Dia. It may be assumed that SO1861 is independently cointernalized within vesicles that contain RIP-loaded SPIONs, and consequently affects the intracellular trafficking and endosomal release of the nanoparticles. This entails that the RIP degradation in the lysosomes is circumvented and thus a greater number of active RIPs are reaching the cytosol to inactivate the ribosomes. As a consequence, the cells enter apoptosis after an enhanced inhibition of the protein synthesis, whereupon cell death occurs [46]. Furthermore, it was examined whether SO1861 maintained the ability to augment the cytotoxicity of RIP-loaded SPIONs even after its binding on the particle surface of SPIONs (SPION-RIP+SPION-SO1861). Self-cytotoxicity of SPION-SO1861 (Supplementary Information, Figure S10) showed no significant toxic effects in the area of interest regarding the toxic profiles of the mixed particle systems, based on equimolar blends of SPION-SO1861 and SPION-Dia or SPION-DiaEGF (Figure 7B). As listed in Table 1, *IC50* values of 2.0 × 10^−6^ M Fe^3+^ and 7.5 × 10^−9^ M Fe^3+^ were determined for SPION-Dia and SPION-DiaEGF in combination with SPION-SO1861, which was less effective than for SPION-RIP+SO1861. The targeting effect of EGF, however, was 267 indicating that SPION-SO1861 more specifically acted on SPION-DiaEGF than free SO1861, presumably due to the adjusted kinetics and uptake mechanisms. The *IC50* values correspond to an enhancement factor of about 270 and 1800, respectively. It is worthwhile to note that the derived *IC50* values were normalized to the amount of RIP-loaded SPIONs present in the respective mixed particle system. In comparison to SPION-RIP+SO1861, SPION-RIP+SPION-SO1861 shows a lower EF. Nevertheless, the cytotoxicity augmentation is still clearly present, evidencing that SO1861 does not forfeit its EEE activity. More importantly, the gain in specificity was higher for SPION-RIP+SPION-SO1861 than for SPION-RIP+SO1861, i.e., the EF ratio for the targeted SPION-DiaEGF compared to the non-targeted SPION-Dia is much greater (6.7 versus 1.6, Table 1), indicating a substantially increased therapeutic window for SPION-RIP+SPION-SO1861 (a ratio of 1.0 corresponds to no widening of the therapeutic window). In addition, it is also important to highlight the lack of a targeting moiety in SPION-SO1861, leading to unspecific cell uptake. The fabrication of SPION-based targeted SO1861 systems thus appears to be a promising approach to further increase the efficacy and prevent off-target effects, which will be investigated in future experiments.

### 3.5. Relaxivity Measurements

**Figure 8:**
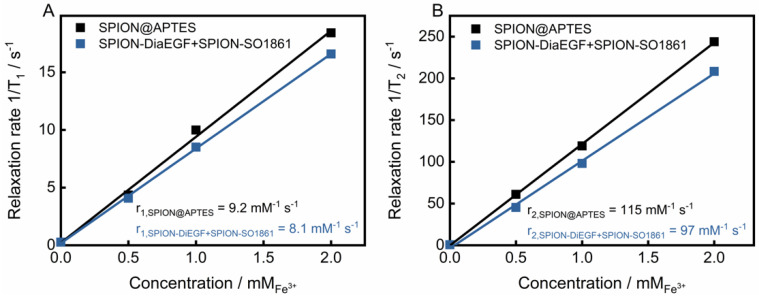
(**A**) T_1_ and (**B**) T_2_ relaxivity measurements of SPION@APTES and the SPION-DiaEGF+ SPION-SO1861 mixture. Values of relaxivites (r_1_ and r_2_) were determined via correlation between the relaxation rates and the corresponding iron concentrations from the slope of the resulting linear regression curve.

Due to their unique magnetic properties, SPIONs can serve not only as drug delivery systems, but also as a diagnostic tool for the detection of cancer via magnetic resonance imaging [47]. Such systems, unifying therapeutic and diagnostic approaches, are referred to as theranostics. To evaluate the potential of the particle mixture of SPION-DiaEGF+SPION-SO1861 to be used as a theranostic system, the longitudinal (T_1_) and transverse (T_2_) relaxation times of protons around the SPIONs were measured. Moreover, the respective relaxivities r_1_ and r_2_, which represent the ability of the SPIONs to alter T_1_ and T_2_, respectively, were determined through the linear fitting of 1/T_1_ or 1/T_2_ versus the iron concentration (Figure 8). As depicted in Figure 8A,B, SPION@APTES are characterized by a low r_1_ (9.2 mM^−1^∙s^−1^) and a high r_2_ (115 mM^−1^∙s^−1^) value. SPIONs predominantly shorten T_2_ relaxation time and thus provide negative contrast in T_2_-weighted images. The relaxivity ratio r_2_/r_1_ further helps to estimate the efficiency of potential T_2_-contrast agents. Here, the ratio was calculated to be 12.5. In comparison, slightly lower relaxivites of r_1_ equal to 8.1 mM^−1^∙s^−1^ and r_2_ equal to 97 mM^−1^∙s^−1^ and thus a ratio of 11.9 was obtained for the mixture of SPION-DiaEGF+SPION-SO1861. Shortening of the relaxation times is, among other reasons, highly related to the surface properties [48]. Hence, the low decrease can be ascribed to a limited magnetic interaction of the SPIONs with the surrounding aqueous medium due to an enhanced steric hindrance as a result of ligand attachment. Nevertheless, the determined values are comparable to those of commercially available SPION-based contrast agents, such as Feridex/Endorem (r_1_ = 27 mM^−1^∙s^−1^, r_2_ = 152 mM^−1^∙s^−1^, r_2_/r_1_ = 5.6) or Resovist (r_1_ = 20.6 mM^−1^∙s^−1^, r_2_ = 86 mM^−1^∙s^−1^, r_2_/r_1_ = 4.2; relaxivities of the commercial contrast agents were measured at 0.47 T in water at 40 °C [49]). Hence, these data demonstrate that the mixture of SPION-DiaEGF+SPION-SO1861 could potentially serve as a theranostic system using magnetic resonance imaging, which illustrates the advantage of SPIONs as a carrier system compared to other particles.

## 4. Conclusions

This study presented the potential impact of glycosylated triterpenoids on SPION-based targeted tumor therapies. For this purpose, SPIONs were synthesized and selectively functionalized with Dia or DiaEGF, by the use of strain-promoted copper-free click chemistry. Extensive characterization of the as-synthesized and -functionalized particle systems showed that the functionalization process resulted in defined SPION-based anti-tumor cell systems without unspecific interparticle or intermolecular crosslinking. Consequently, in vitro studies on colon carcinoma cell lines showed that the enzymatic activity of dianthin and the targeting ability of EGF were preserved. The number of functionalized particles required to elicit the toxic effect was tremendously reduced in a combinatorial approach with the addition of free SO1861 by an enhancement factor of 21000 and 33000 for dianthin and dianthin-EGF-modified SPIONs, respectively. Finally, SO1861 was also coordinated on the SPION surface in order to unify the disparate pharmacokinetics of the single components, dianthin-EGF and SO1861. In this way, cytotoxicity was enhanced 270 and 1800 times after applying SPION-Dia or SPION-DiaEGF combined with SPION-SO1861, thus resulting in a substantial widening of the therapeutic window in cell culture. Accordingly, the efficacy of the nanoparticulate formulations was substantially improved, which facilitates the acceptance of this drug delivery system from a clinical perspective. Furthermore, relaxivity measurements of the combined particle mixture of SPION-DiaEGF and SPION-SO1861 have shown that the utilized particles could potentially serve for theranostic approaches. This study thereby offers new opportunities for the design of novel nanoparticle-based anti-tumor agents by reducing the effective drug concentration and thus possible side effects of tumor therapies.

## Figures and Tables

**Table 1:** Corresponding half maximal inhibitory concentration (*IC50*) values and enhancement factor (*EF*) derived from Figure 6 and Figure 7 of the respective particle based anti-tumor systems. Since the *IC50* was not reached for SPION@APTES and SPION@BCN, specification of the *EF* was not possible for these particle systems. The targeting effect is defined as the ratio of the *IC50* values of the non-targeted compound (SPION-Dia) to the targeted compound (SPION-DiaEGF). The gain in specificity is the ratio of the targeted *EF* to the non-targeted *EF*.

	*w/o* SO1861/M	+SO1861/M	*EF*	+SPION-SO1861/M	*EF*
**SPION@APTES**	>2 × 10^−3^	4.5 × 10^−5^	–	2.2 × 10^−5^	–
**SPION-BCN**	>2 × 10^−3^	–	–	–	–
**SPION-Dia**	5.5 × 10^−4^	2.6 × 10^−8^	21,000	2.0 × 10^−6^	270
**SPION-DiaEGF**	1.3 × 10^−5^	4.0 × 10^−10^	33,000	7.5 × 10^−9^	1800
**Targeting Effect**	42	65	–	267	–
**Gain in Specificity**	–	–	1.6	–	6.7

## Data Availability

Data are available upon request.

## Supplementary Materials

The following are available online at https://www.mdpi.com/article/10.3390/nano11041057/s1, additional technical details regarding the XRD measurement, Figure S1: Schematic representation of all modification steps, Figure S2: SDS/PAGE (12%) of the purified fractions of Dia and DiaEGF obtained after Ni-NTA and chitin column tandem affinity chromatography, Figure S3: MALDI-TOF-MS spectra of Dia and conjugates with different molar ratios of linker:Dia, Figure S4: MALDI-TOF-MS spectra of DiaEGF and conjugates obtained from different initial molar ratios of linker:DiaEGF in the reaction mixture, Figure S5: XRD-Diffractogram of synthesized SPIONs and reference pattern of magnetite/maghemite, Figure S6: FTIR spectra of SPIONs after each functionalization step, Figure S7: DLS measurement of SPION-SO1861 revealing a median size of 84 nm, Figure S8: Cytotoxicity studies of Dia and DiaEGF conjugates on EGFR-overexpressing HCT-116 cells, Figure S9: Self-cytotoxicity of SO1861 on HCT-116. The derived *IC50* value accounts for 3.3 μg/mL, Figure S10: Self-cytotoxicity of SPION-SO1861 on HCT-116. The *IC50* value is determined as >1 mM Fe^3+^.

## References

[1] Ferlay J., Colombet M., Soerjomataram I., Mathers C., Parkin D.M., Piñeros M., Znaor A., Bray F. (2019). Estimating the global cancer incidence and mortality in 2018: GLOBOCAN sources and methods. Int. J. Cancer.

[2] Pastan I., Hassan R., Fitzgerald D.J., Kreitman R.J. (2006). Immunotoxin therapy of cancer. Nat. Rev. Cancer.

[3] Koo O.M., Rubinstein I., Onyuksel H. (2005). Role of nanotechnology in targeted drug delivery and imaging: A concise review. Nanomedicine.

[4] Arteaga C.L. (2002). Epidermal growth factor receptor dependence in human tumors: More than just expression?. Oncologist.

[5] Salomon D.S., Brandt R., Ciardiello F., Normanno N. (1995). Epidermal growth factor-related peptides and their receptors in human malignancies. Crit. Rev. Oncol. Hematol..

[6] Rocha-Lima C.M., Soares H.P., Raez L.E., Singal R. (2007). EGFR targeting of solid tumors. Cancer Control.

[7] Simon N., FitzGerald D. (2016). Immunotoxin Therapies for the Treatment of Epidermal Growth Factor Receptor-Dependent Cancers. Toxins.

[8] Wykosky J., Fenton T., Furnari F., Cavenee W.K. (2011). Therapeutic targeting of epidermal growth factor receptor in human cancer: Successes and limitations. Chin. J. Cancer.

[9] Canton I., Battaglia G. (2012). Endocytosis at the nanoscale. Chem. Soc. Rev..

[10] Puri M., Kaur I., Perugini M.A., Gupta R.C. (2012). Ribosome-inactivating proteins: Current status and biomedical applications. Drug Discov. Today.

[11] Smith S.A., Selby L.I., Johnston A.P.R., Such G.K. (2019). The Endosomal Escape of Nanoparticles: Toward More Efficient Cellular Delivery. Bioconjugate Chem..

[12] Bhargava C., Dürkop H., Zhao X., Weng A., Melzig M.F., Fuchs H. (2017). Targeted dianthin is a powerful toxin to treat pancreatic carcinoma when applied in combination with the glycosylated triterpene SO1861. Mol. Oncol..

[13] Fuchs H., Niesler N., Trautner A., Sama S., Jerz G., Panjideh H., Weng A. (2017). Glycosylated Triterpenoids as Endosomal Escape Enhancers in Targeted Tumor Therapies. Biomedicines.

[14] Baluna R., Vitetta E.S. (1997). Vascular leak syndrome: A side effect of immunotherapy. Immunopharmacology.

[15] Kuus-Reichel K., Grauer L.S., Karavodin L.M., Knott C., Krusemeier M., Kay N.E. (1994). Will immunogenicity limit the use, efficacy, and future development of therapeutic monoclonal antibodies?. Clin Diagn Lab Immunol.

[16] Selbo P.K., Bostad M., Olsen C.E., Edwards V.T., Høgset A., Weyergang A., Berg K. (2015). Photochemical internalisation, a minimally invasive strategy for light-controlled endosomal escape of cancer stem cell-targeting therapeutics. Photochem. Photobiol. Sci..

[17] Zhang D., Wang J., Xu D. (2016). Cell-penetrating peptides as noninvasive transmembrane vectors for the development of novel multifunctional drug-delivery systems. J. Control. Release.

[18] Fuchs H., Bachran C., Flavell D. (2013). Diving through Membranes: Molecular Cunning to Enforce the Endosomal Escape of Antibody-Targeted Anti-Tumor Toxins. Antibodies.

[19] Ag Seleci D., Seleci M., Stahl F., Scheper T. (2017). Tumor homing and penetrating peptide-conjugated niosomes as multi-drug carriers for tumor-targeted drug delivery. RSC Adv..

[20] Bachran C., Bachran S., Sutherland M., Bachran D., Fuchs H., Atta-ur-Rahman, Choudhary M.I., Perry G. (2014). Preclinical Studies of Saponins for Tumor Therapy. Recent Advances in Medicinal Chemistry.

[21] Fuchs H., Bachran D., Panjideh H., Schellmann N., Weng A., Melzig M.F., Sutherland M., Bachran C. (2009). Saponins as tool for improved targeted tumor therapies. Curr. Drug Targets.

[22] Bachran C., Bachran S., Sutherland M., Bachran D., Fuchs H. (2008). Saponins in tumor therapy. Mini-Rev. Med. Chem..

[23] Gilabert-Oriol R., Mergel K., Thakur M., Mallinckrodt B., von Melzig M.F., Fuchs H., Weng A. (2013). Real-time analysis of membrane permeabilizing effects of oleanane saponins. Bioorganic & medicinal chemistry.

[24] Mallinckrodt B., von Thakur M., Weng A., Gilabert-Oriol R., Dürkop H., Brenner W., Lukas M., Beindorff N., Melzig M.F., Fuchs H. (2014). Dianthin-EGF is an effective tumor targeted toxin in combination with saponins in a xenograft model for colon carcinoma. Future Oncol..

[25] Weng A., Thakur M., Beceren-Braun F., Bachran D., Bachran C., Riese S.B., Jenett-Siems K., Gilabert-Oriol R., Melzig M.F., Fuchs H. (2012). The toxin component of targeted anti-tumor toxins determines their efficacy increase by saponins. Mol. Oncol..

[26] Bachran C., Weng A., Bachran D., Riese S.B., Schellmann N., Melzig M.F., Fuchs H. (2010). The distribution of saponins in vivo affects their synergy with chimeric toxins against tumours expressing human epidermal growth factor receptors in mice. Br. J. Pharmacol..

[27] Choi H.S., Liu W., Liu F., Nasr K., Misra P., Bawendi M.G., Frangioni J.V. (2010). Design considerations for tumour-targeted nanoparticles. Nat. Nanotechnol..

[28] Ling D., Hyeon T. (2013). Chemical design of biocompatible iron oxide nanoparticles for medical applications. Small.

[29] Bourrinet P., Bengele H.H., Bonnemain B., Dencausse A., Idee J.-M., Jacobs P.M., Lewis J.M. (2006). Preclinical safety and pharmacokinetic profile of ferumoxtran-10, an ultrasmall superparamagnetic iron oxide magnetic resonance contrast agent. Investig. Radiol..

[30] Patsula V., Moskvin M., Dutz S., Horák D. (2016). Size-dependent magnetic properties of iron oxide nanoparticles. J. Phys. Chem. Solids.

[31] Ma D. (2014). Enhancing endosomal escape for nanoparticle mediated siRNA delivery. Nanoscale.

[32] Maurer V., Frank C., Porsiel J.C., Zellmer S., Garnweitner G., Stosch R. (2020). Step-by-step monitoring of a magnetic and SERS-active immunosensor assembly for purification and detection of tau protein. J. Biophotonics.

[33] Asadian-Birjand M., Biglione C., Bergueiro J., Cappelletti A., Rahane C., Chate G., Khandare J., Klemke B., Strumia M.C., Calderón M. (2016). Transferrin Decorated Thermoresponsive Nanogels as Magnetic Trap Devices for Circulating Tumor Cells. Macromol. Rapid Commun..

[34] Gilabert-Oriol R., Thakur M., Weise C., Dernedde J., Mallinckrodt B., von Fuchs H., Weng A. (2013). Small structural differences of targeted anti-tumor toxins result in strong variation of protein expression. Protein Expr. Purif..

[35] Gilabert-Oriol R., Weng A., Trautner A., Weise C., Schmid D., Bhargava C., Niesler N., Wookey P.J., Fuchs H., Thakur M. (2015). Combinatorial approach to increase efficacy of Cetuximab, Panitumumab and Trastuzumab by dianthin conjugation and co-application of SO1861. Biochem. Pharmacol..

[36] Gilabert-Oriol R., Thakur M., Haussmann K., Niesler N., Bhargava C., Görick C., Fuchs H., Weng A. (2016). Saponins from Saponaria officinalis L. Augment the Efficacy of a Rituximab-Immunotoxin. Planta Med..

[37] Balin-Gauthier D., Delord J.-P., Rochaix P., Mallard V., Thomas F., Hennebelle I., Bugat R., Canal P., Allal C. (2006). In vivo and in vitro antitumor activity of oxaliplatin in combination with cetuximab in human colorectal tumor cell lines expressing different level of EGFR. Cancer Chemother. Pharmacol..

[38] Anido J., Matar P., Albanell J., Guzmán M., Rojo F., Arribas J., Averbuch S., Baselga J. (2003). ZD1839, a specific epidermal growth factor receptor (EGFR) tyrosine kinase inhibitor, induces the formation of inactive EGFR/HER2 and EGFR/HER3 heterodimers and prevents heregulin signaling in HER2-overexpressing breast cancer cells. Clin. Cancer Res..

[39] Zarinwall A., Waniek T., Saadat R., Braun U., Sturm H., Garnweitner G. Comprehensive Characterization of APTES Surface Modifications of Hydrous Boehmite Nanoparticles. Langmuir.

[40] Fuchs H. (2019). Dianthin and Its Potential in Targeted Tumor Therapies. Toxins.

[41] Weng A. (2018). A novel adenine-releasing assay for ribosome-inactivating proteins. J. Chromatogr. B.

[42] Masthoff I.-C., Kraken M., Mauch D., Menzel D., Munevar J.A., Baggio Saitovitch E., Litterst F.J., Garnweitner G. (2014). Study of the growth process of magnetic nanoparticles obtained via the non-aqueous sol–gel method. J. Mater. Sci..

[43] Park C., Vo C.L.-N., Kang T., Oh E., Lee B.-J. (2015). New method and characterization of self-assembled gelatin-oleic nanoparticles using a desolvation method via carbodiimide/N-hydroxysuccinimide (EDC/NHS) reaction. Eur. J. Pharm. Biopharm..

[44] Vranic S., Gatalica Z., Wang Z.-Y. (2011). Update on the molecular profile of the MDA-MB-453 cell line as a model for apocrine breast carcinoma studies. Oncol. Lett..

[45] Bachran D., Schneider S., Bachran C., Urban R., Weng A., Melzig M.F., Hoffmann C., Kaufmann A.M., Fuchs H. (2010). Epidermal growth factor receptor expression affects the efficacy of the combined application of saponin and a targeted toxin on human cervical carcinoma cells. Int. J. Cancer.

[46] Wayne A.S., Fitzgerald D.J., Kreitman R.J., Pastan I. (2014). Immunotoxins for leukemia. Blood.

[47] Rosen J.E., Chan L., Shieh D.-B., Gu F.X. (2012). Iron oxide nanoparticles for targeted cancer imaging and diagnostics. Nanomedicine.

[48] Huang J., Zhong X., Wang L., Yang L., Mao H. (2012). Improving the magnetic resonance imaging contrast and detection methods with engineered magnetic nanoparticles. Theranostics.

[49] Rohrer M., Bauer H., Mintorovitch J., Requardt M., Weinmann H.-J. (2005). Comparison of magnetic properties of MRI contrast media solutions at different magnetic field strengths. Investig. Radiol..

