# Supplementary material for: Magnetic Nanoparticle-Based Dianthin Targeting for Controlled Drug Release Using the Endosomal Escape Enhancer SO1861
Source: Nanomaterials (Basel). 2021 Apr 20;11(4):1057. doi: 10.3390/nano11041057 (PMC8074366; doi:10.3390/nano11041057)
Supplement: Supplementary file 1 [file nanomaterials-11-01057-s001.zip › nanomaterials-1166493-supplementary.pdf]

Magnetic Nanoparticle-based Dianthin Targeting for Controlled Drug Release using the Endosomal Escape Enhancer SO1861

A. Zarinwall[1,2,3], M. Asadian-Birjand[3], D. Ag Seleci[1,2], V. Maurer[1,2], Alexandra Trautner[3], G. Garnweitner[1,2] & H. Fuchs[3]*

[1] Institute for Particle Technology (iPAT) | Technische Universität Braunschweig | 38104 Braunschweig, Germany

[2] Center of Pharmaceutical Engineering Research (PVZ) | Technische Universität Braunschweig | 38106 Braunschweig, Germany

[3] Charité – Universitätsmedizin Berlin, corporate member of Freie Universität Berlin and Humboldt-Universität zu Berlin | Institute of Laboratory Medicine, Clinical Chemistry and Pathobiochemistry | 13353 Berlin, Germany

[*] correspondence to: g.garnweitner@tu-braunschweig.de

Table of Content

1. Additional technical details
2. Figure S1: Schematic representation of each modification step
3. Figure S2: SDS/PAGE electrophoresis of Dia and DiaEGF
4. Figure S3: MALDI-TOF spectra of Dia
5. Figure S4: MALDI-TOF spectra of DiaEGF
6. Figure S5: XRD diffractogram of SPIONs
7. Figure S6: FTIR spectra of SPIONs after each step of functionalization
8. Figure S7: DLS measurement of SPION-SO1861
9. Figure S8: Cytotoxicity studies of Dia-PEG_{12}-N_3 and DiaEGF-PEG_{12}-N_3 on HCT-116
10. Figure S9: Self-cytotoxicity of SO1861 on HCT-116
11. Figure S10: Self-cytotoxicity of SPION-SO1861 on HCT-116

Additional technical details

X-ray diffraction (XRD) was performed on an Empyrean system from Malvern Panalytical with Cu Kα radiation on a Si sample holder in a range of 2θ from 20 to 90° and a step size of 0.05°. Evaluation was accomplished by database research using the Inorganic Crystal Structure Database (ICSD).

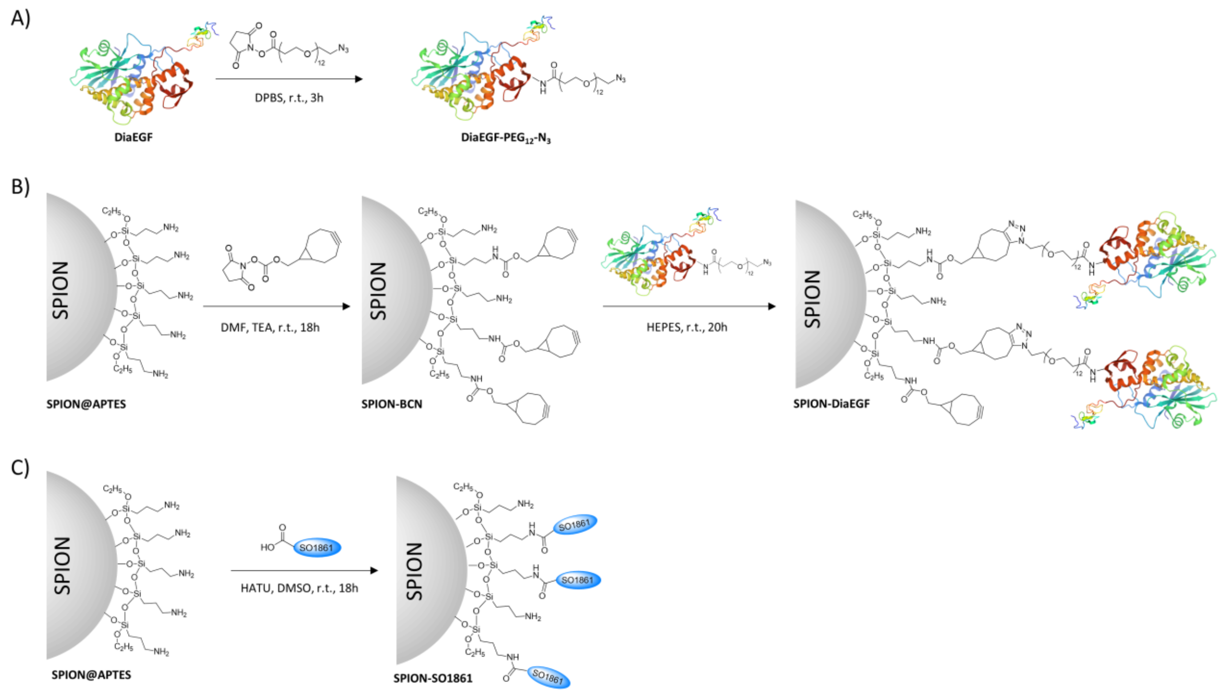

Figure S1. Schematic representation of the respective modification steps: A) Conjugation of the NHS-PEG12-N3 linker to DiaEGF; B) functionalization of APTES-modified SPIONs (SPION@APTES) with NHS-BCN followed by DiaEGF-PEG12-N3; C) Coordination of SO1861 on SPION@APTES.

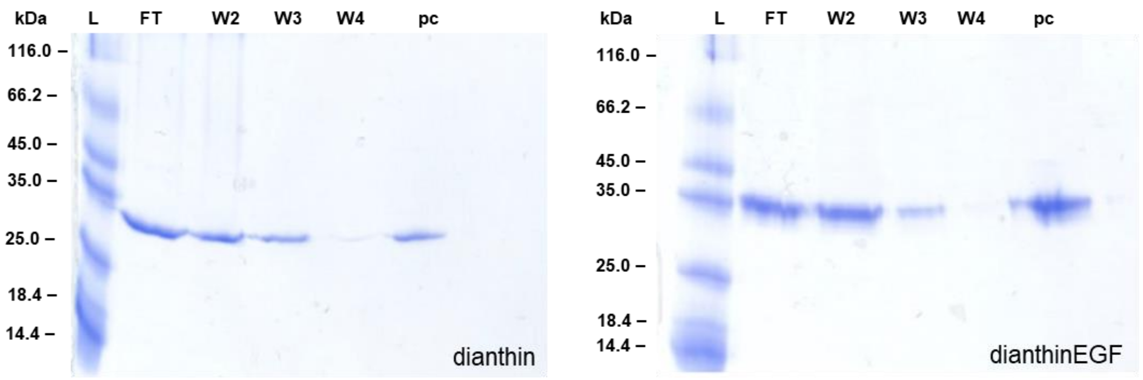

Figure S2. SDS/PAGE (12%) of the purified fractions of Dia and DiaEGF obtained after Ni-NTA and chitin column tandem affinity chromatography. L, Ladder; FT, flow-through; W, washout; pc, positive control (confirmed previous batch).

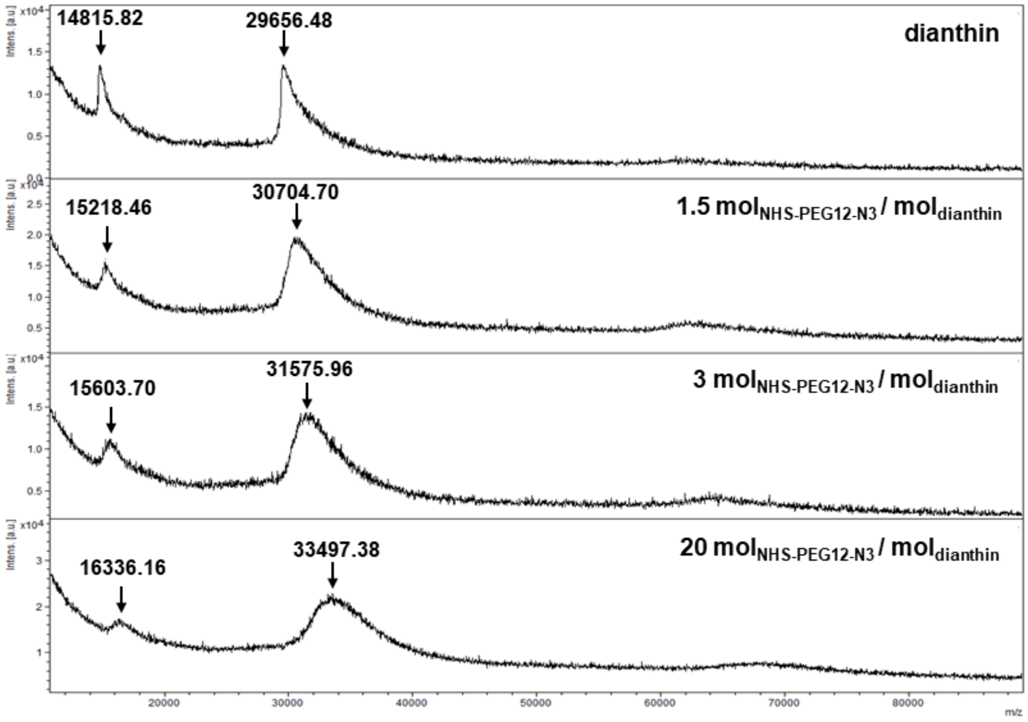

Figure S3. MALDI-TOF-MS spectra of Dia and conjugates with different molar ratios of linker:Dia.

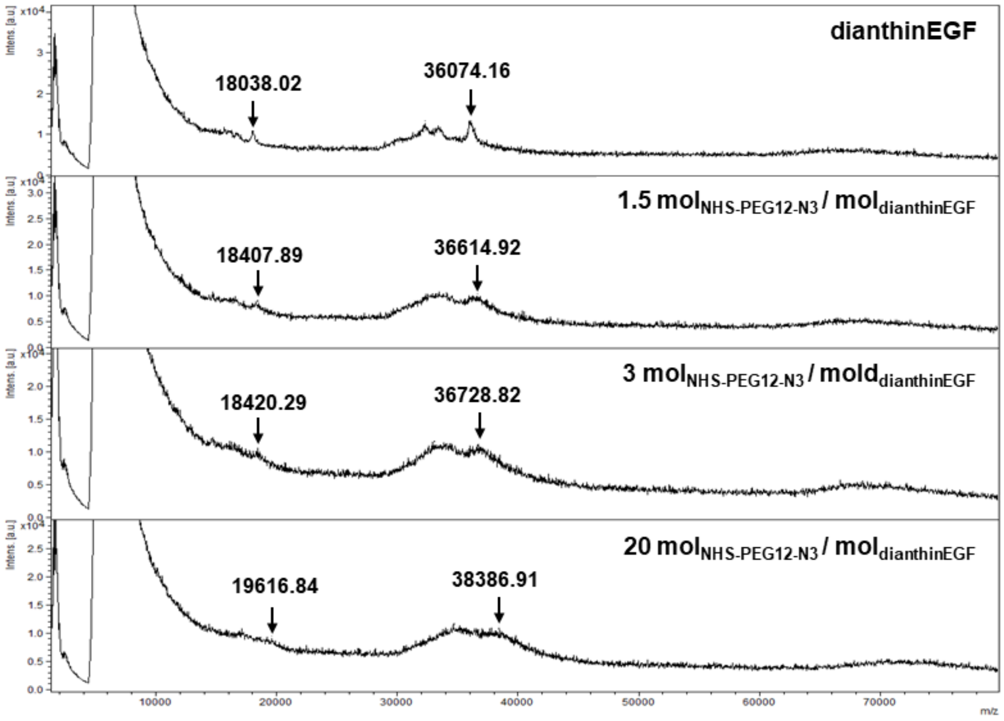

Figure S4. MALDI-TOF-MS spectra of DiaEGF and conjugates obtained from different initial molar ratios of linker:DiaEGF in the reaction mixture. The peaks appearing at approximately 32730 g/mol are attributable to degradation products of DiaEGF.

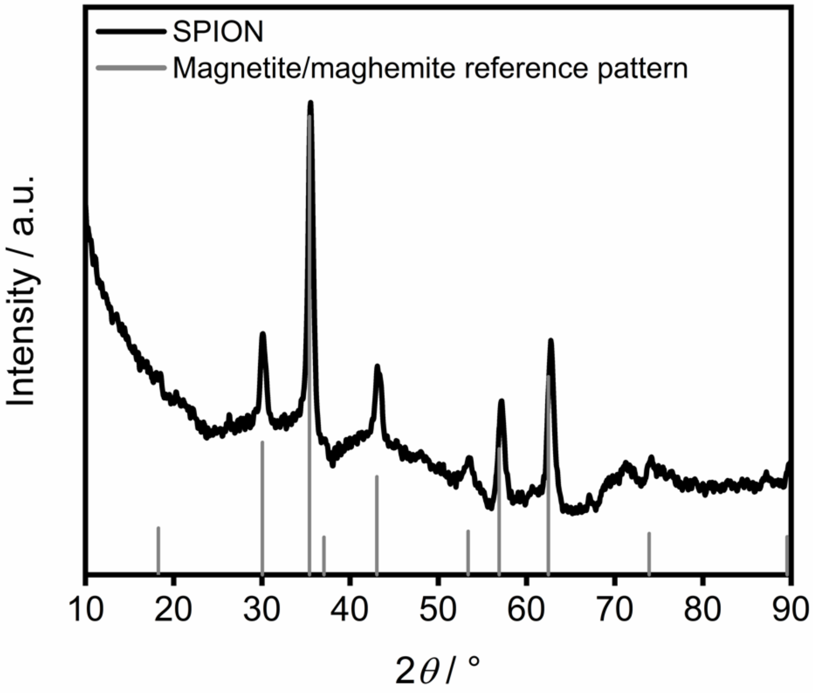

Figure S5. X-ray diffractogram of synthesized SPIONs and reference pattern of magnetite/maghemite. The initial decrease in the curve implies an amorphous fraction on the particle surface. According to the inorganic crystal structure database (ICSD 98-002-0596), the reflections obtained at 30°, 35°, 43°, 57° and 62° are attributable to a mixed crystalline phase of magnetite and maghemite. Applying the Debye-Scherrer equation to the highest intensity reflection at 35.5° with a full width at half maximum (FWHM) of 0.7°, KS of 0.9 and wavelength λ of 0.154 nm, a crystallite size of 12.8 nm was determined.

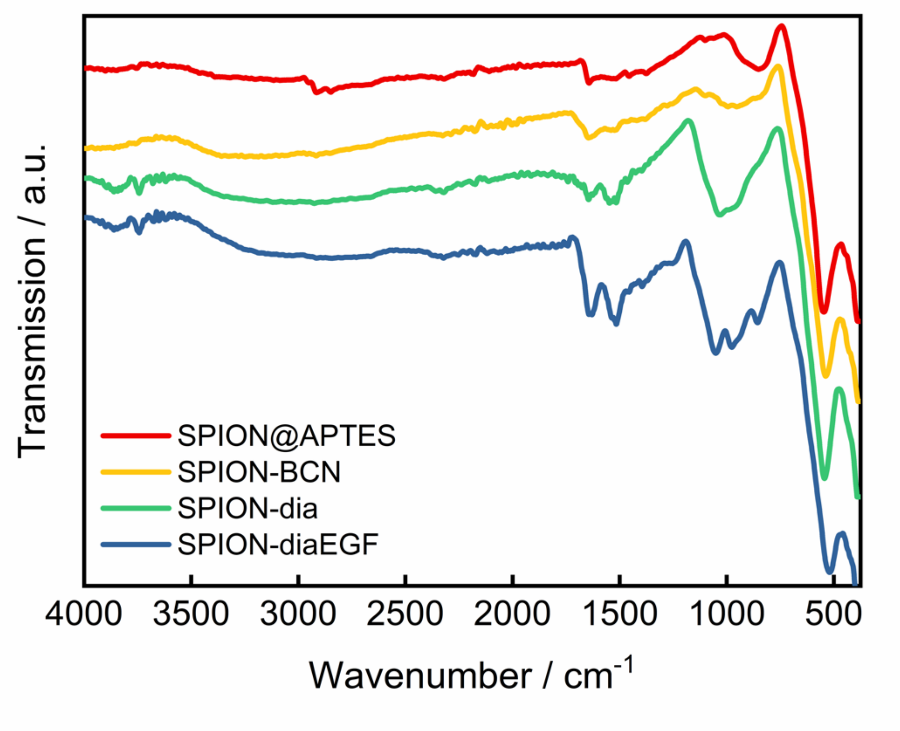

Figure S6. FTIR spectra of SPIONs after each functionalization step.

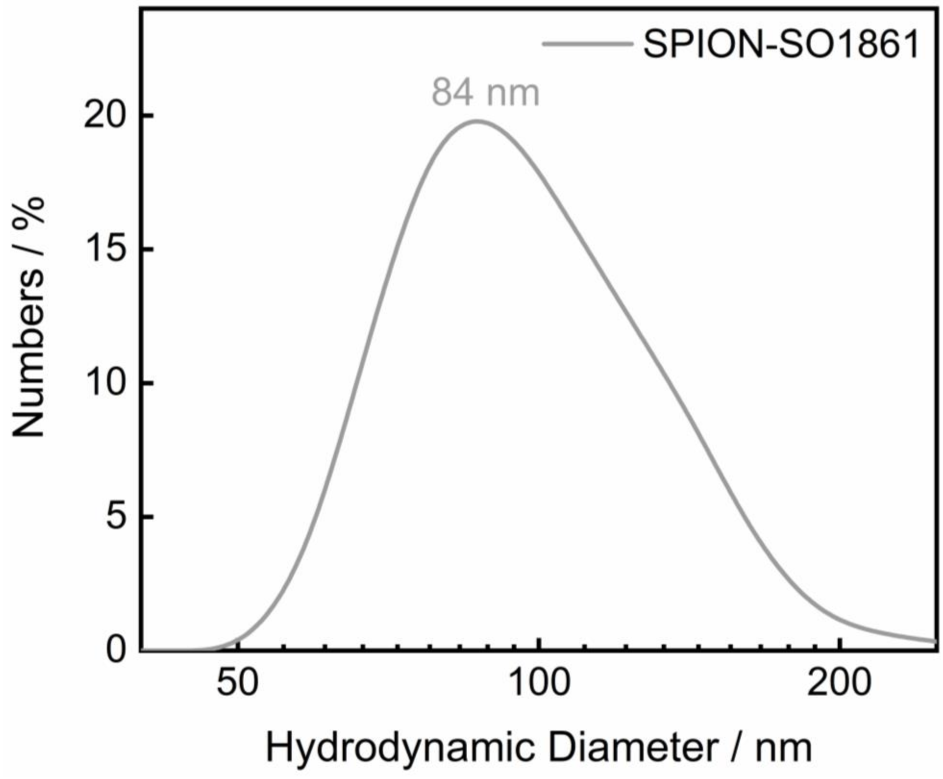

Figure S7. DLS measurement of SPION-SO1861 revealing a median size of 84 nm.

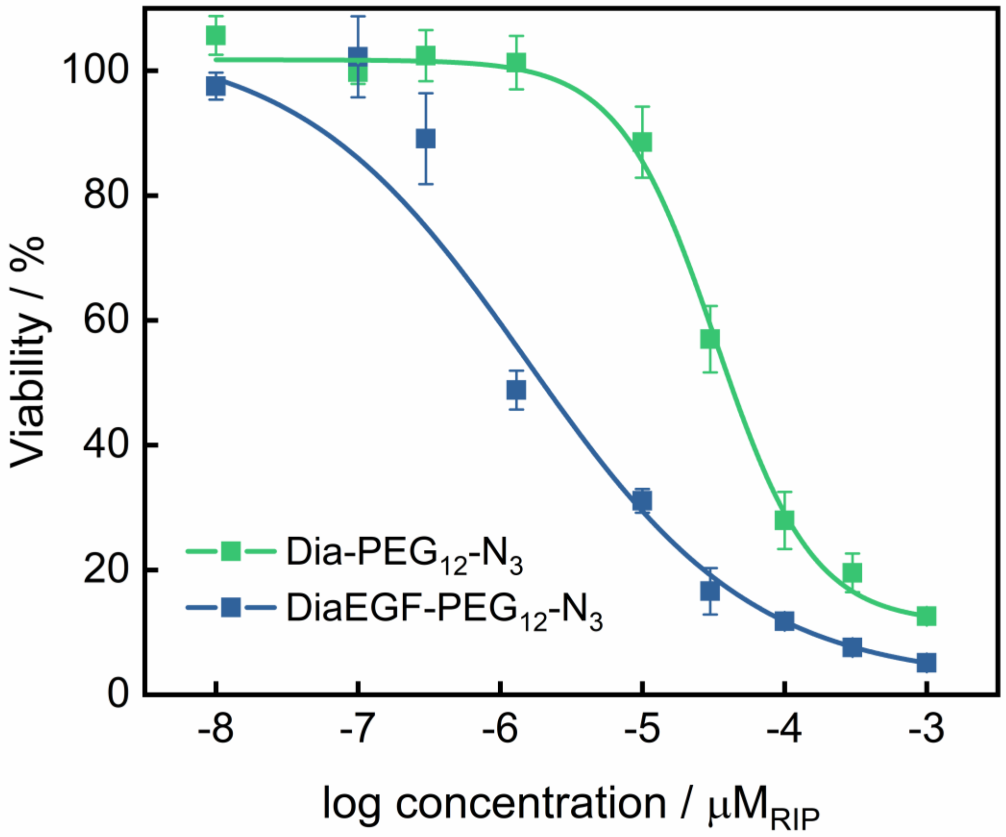

Figure S8. Cytotoxicity studies of Dia and DiaEGF conjugates on EGFR-overexpressing HCT-116 cells. Cell viability was assessed after 48 h by MTT-assay and plotted as a function of the RIP concentration. The experiment was performed in quadruplicates. Accordingly, Dia-PEG$_{12}$-N$_3$ and DiaEGF-PEG$_{12}$-N$_3$ have an IC_{50} value of $4.3*10^{-5}$ M and $1.2*10^{-6}$ µM, respectively.

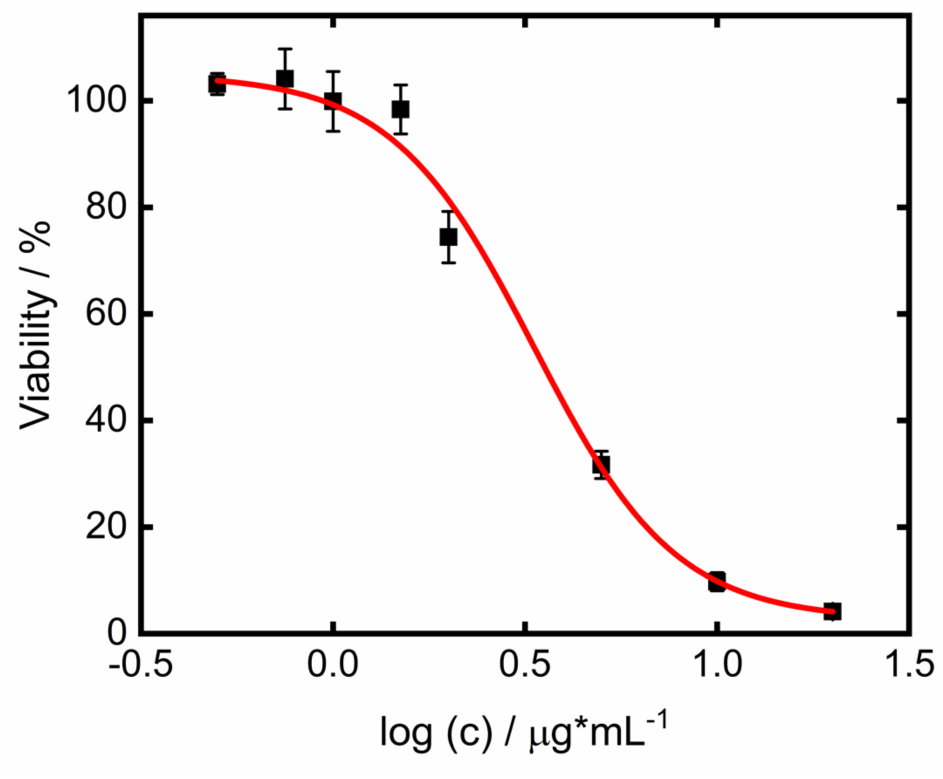

Figure S9. Self-cytotoxicity of SO1861 on HCT-116. The derived IC_{50} value accounts for 3.3 μg/mL.

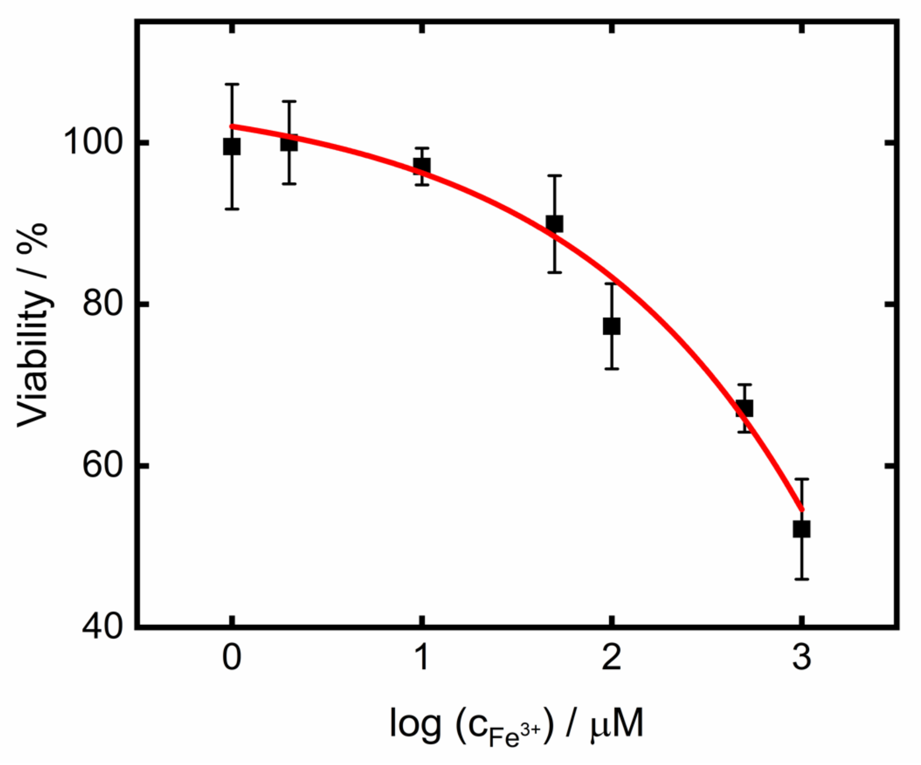

Figure S10. Self-cytotoxicity of SPION-SO1861 on HCT-116. The IC_{50} value is determined as > 1 mM Fe^{3+}.
